# Enlargements of the Liver.—III

**Published:** 1910-01-15

**Authors:** 


### Medicine.

### ENLARGEMENTS OF THE LIVER.—III.

A fatty liver may be only slightly enlarged, or it may be bulky and uniformly increased in size. Although it may weigh many ounces more than a normal liver, its specific gravity may be so diminished that slices of the organ may float in water. The surface is smooth and soft and the edge either natural or somewhat rounded. The condition may be associated with general obesity, or with toxic maladies such as acute fevers, alcoholism, phthisis, and other wasting diseases, phorphorus poisoning, diabetic acidosis, chloroform toxæmia, and so forth. A physical examination reveals that the liver is enlarged and painless, the surface smooth, and the edge indefinite in form or rounded. The symptoms are usually those of the associated condition. There is neither pain, jaundice, ascites, nor enlargement of the spleen.

### The Liver in Lardaceous Disease.

A lardaceous liver is not necessarily enlarged, but it may be considerably or even enormously bigger than normal. It may weigh as much as 14 lbs. (Wilks). It is pale, hard, firm, and inelastic. The edge may be sharp and firm, or if there is also fatty degeneration, somewhat rounded. On section it has a peculiar translucent, soapy appearance, and it cuts like raw bacon. It is comparatively dry. The specific gravity is increased.

A physical examination reveals a large, hard, firm tumour with the characteristic signs of an enlargement of the liver, with a smooth surface and a sharp, well-defined or hard-rounded edge. There is neither pain nor tenderness on palpating it. It is usually associated with enlargement of the spleen, owing to lardaceous change in the latter also; with albuminuria if the kidneys are affected; and diarrhœa if the intestines are involved. The patient is usually anæmic. Neither jaundice nor ascites occurs as a direct result of the lardaceous change.

### Catarrhal and Obstructive Jaundice.

Whenever there is an obstruction to the free outflow of bile from the liver, not only does jaundice develop, but the liver becomes more or less enlarged and tender, the edge becoming palpable below the costal margin in the right nipple line.

Catarrhal jaundice occurs most frequently in young, healthy subjects; it is not accompanied by pain, it is not associated with emaciation, and a physical examination is of a negative character, as far as cirrhosis, carcinoma or suppuration of the liver are concerned. The gall-bladder does not enlarge.

In obstructive jaundice, due to calculus, there may be a history of attacks of biliary colic, characterised by sudden agonising pain in the right hypochondrium, going through to the back of the chest and up to the back of the right shoulder, rigors, shuddering, profuse sweating, pyrexia, and even collapse, with rapid, feeble pulse.

Chronic pancreatitis is a disease whose symptoms closely simulate those of gall-stones with recurrent colic. Owing to the catarrh in the pancreatic duct extending to the ampulla of Vater and there involving the common opening of bile duct and pancreatic duct, obstructive jaundice develops in varying degree. Natural attempts to overcome the catarrhal obstruction lead to colicky attacks of pain. The liver enlarges just as it may do from any other variety of biliary obstruction. As regards the gall-bladder, it is more liable to enlarge in cases of chronic pancreatitis than it is with gall-stones. Cammidge's urine test may assist in the differential diagnosis. The chief importance of distinguishing between gall-stones and chronic pancreatitis is in connection with the operative treatment of the former. Urotropine is a drug well worthy of trial in either case.

Carcinoma of the head of the pancreas may give rise to deepening jaundice, hepatic enlargement, distension of the gall-bladder, with little else in the way of symptoms. The diagnosis is arrived at partly by a process of exclusion, partly by watching the course of the disease, particular stress being laid upon the liability of the gall-bladder to exhibit great distension in some of these cases.

### Some Rarer Liver Lesions.

Suppurative pylephlebitis is fortunately not a common disease, but it is of importance, because when it does occur it is seldom diagnosed, and it is responsible for very baffling symptoms. It is an inflammation of the portal vein associated with the formation of suppurating thrombi in the branches of this vessel within the liver; it is one of the causes of multiple disseminated abscesses in the liver. It is nearly always secondary to inflammation in or around a viscus whose blood drains into the portal vein; it may therefore be caused by inflammatory or ulcerative affections of the alimentary canal, from gastric ulcer to piles. Its commonest cause by far is appendicitis, and it follows mild attacks of the latter rather than severe ones.

In more than half the cases the liver is definitely and uniformly enlarged. There may be a slight fulness in the right hypochondriac and epigastric regions. The edge of the liver may be felt just below the costal margin, and occasionally as low as the umbilicus; it is sharp and well defined. The surface is smooth, regular, and tender. The lower part of the thorax on the right side may be bulged, and the intercostal spaces there filled out as the result of perihepatitis. The chief symptoms are pyrexia, rigors, sweating, prostration, emanciation, sallow earthy complexion, abdominal pain, and tenderness in the right hypochondriac and epigastric regions, jaundice in a few instances, and some enlargement of the liver; the association of these with, or their following after any ulcerative or inflammatory lesion of the intestinal tract, particularly the appendix vermiformis, should suggest the possibility of suppurative pylephlebitis.

*(To be continued.)*

